# A simple and learning-friendly “Chinese technique” for arthroscopic PCL reconstruction with remnant preservation: all-anterior aproach

**DOI:** 10.3389/fsurg.2025.1682950

**Published:** 2025-11-06

**Authors:** Gamal A. Elsawy

**Affiliations:** Orthopedic Surgery Department, Al-Azhar University, Cairo, Egypt

**Keywords:** posterior cruciate ligament, reconstruction, arthroscopy, all-anterior approach, Chinese technique

## Introduction

Posterior cruciate ligament (PCL) injury present a significant challenge in knee surgery. They often occur in high-energy events and can lead to chronic instability if not properly managed. Over the years, various arthroscopic techniques have been developed for PCL reconstruction. The traditional transtibial technique involves an oblique tunnel through the tibia. However, this method has well-recognized limitations: visualization of the PCL insertion is poor through standard anterior portals, the tibial tunnel is often placed non-anatomically, and the graft has a sharp turn (the “killer turn”) when it enter the joint (1). This acute angle can abrade and weaken the graft. Therefore, novel techniques are explored over time.

Last month I travelled to Shanghai, China to visit of Prof. Jiwu Chen and his team for an exchange learning in arthroscopic techniques in sports medicine. One of the shocking points is the all-anterior arthroscopic PCL reconstruction (detailed elsewhere (2), a simple Chinese Technique with less iatrogenic lesion compared to other PCL reconstruction techniques.

## A patient-friendly technique

An important feature of this novel technique is remnant presevation and the avoidance of killer turn. Both are pivotal to secure a low failure rate. In traditional PCL reconstructions, the remnant tissue is often debrided to improve visualization of the insertion and to make space for the implantation. In contrast, the all-anterior approach is explicitly remnant-preserving, aiming to retain as much of the native PCL tissue as possible. The remnant is not simply left in place haphazardly; rather, it is carefully mobilized from its avulsed position on the tibia and repositioned so that it lies alongside the new graft. This preserved remnant can provide several biological advantages.

Firstly, the PCL remnant contains viable cells and matrix that may contribute to graft healing and integration. There is evidence that preserving remnant fibers in cruciate ligament surgery can enhance the biological incorporation of the graft. In ACL reconstruction, remnant preservation has been associated with improved graft revascularization and better proprioceptive function (3). Regarding PCL, the principle is thought to be similar. The remnant tissue can act as a scaffold that promotes graft incorporation and may even help in maintaining the normal anatomic course of the ligament.

Secondly, preserving the remnant can improve the mechanics of the reconstructed knee. The native PCL fibers, if left attached to the femur, can still contribute some stength, especially in the early postoperative period. Some surgeons have even advocated for techniques that augment the PCL by repairing the remnant in addition to reconstruction in chronic tears. In the all-anterior technique, the graft is deliberately passed over the remnant as it goes from tibia to femur. The remnant acts as a natural cushion, reducing friction and wear on the graft at the critical point where it turns into the joint, thus mitigating the desarstrous impact of killer-turn on graft survival.

In addition, preserving the remnant may have proprioceptive benefits. The PCL, like other ligaments, contains mechanoreceptors that contribute to joint stability sense (4). Removing the remnant would eliminate these receptors, making it possible for an early recovery and returning to sports, which is especially important for athletes.

## A learning-friendly technique

An apparent advantage of this technique is the simplification of the surgical procedure. By eliminating the need for posterior portals or separate incision as required in other techniques, the all-anterior technique reduces the number of steps and instruments involved, thus shortening not only the surgical time but also the learning curve (5). Yaying Sun, the member of Prof. Chen team in charge of teaching and training for students and fellows, gives explanations on key steps. Even post-graduate students with limited clinical experience can easily get the principle of this operation quickly.

## Discussion

The all-anterior approach for anthroscopic PCL reconstruction is an innovative technique. Different from other PCL reconstruction techniques, this procedure is simple and easy to learn. Shortened surgery time and learning curve make it both surgeon-friendly and patient-friendly. All patients can stand up and walk with the help of brace one day after surgery. Long-term follow-up data and comparisons to other clinical trials with different techniques are expected to further validate the application of this procedure.
